# Radiomics Analysis of Multi-Sequence MR Images For Predicting Microsatellite Instability Status Preoperatively in Rectal Cancer

**DOI:** 10.3389/fonc.2021.697497

**Published:** 2021-07-07

**Authors:** Zongbao Li, Hui Dai, Yunxia Liu, Feng Pan, Yanyan Yang, Mengchao Zhang

**Affiliations:** ^1^ China-Japan Union Hospital of Jilin University, Changchun, China; ^2^ The First Affiliated Hospital of Soochow University, Suzhou, China

**Keywords:** magnetic resonance, rectal cancer, microsatellite instability, radiomics, multi-sequence MR

## Abstract

**Background:**

Immunotherapy, adjuvant chemotherapy, and prognosis of colorectal cancer are associated with MSI. Biopsy pathology cannot fully reflect the MSI status and heterogeneity of rectal cancer.

**Purpose:**

To develop a radiomic-based model to preoperatively predict MSI status in rectal cancer on MRI.

**Assessment:**

The patients were divided into two cohorts (training and testing) at a 7:3 ratio. Radiomics features, including intensity, texture, and shape, were extracted from the segmented volumes of interest based on T2-weighted and ADC imaging.

**Statistical Tests:**

Independent sample t test, Mann-Whitney test, the chi-squared test, Receiver operating characteristic curves, calibration curves, decision curve analysis and multi-variate logistic regression analysis

**Results:**

The radiomics models were significantly associated with MSI status. The T2-based model showed an area under the curve of 0.870 with 95% CI: 0.794–0.945 (accuracy, 0.845; specificity, 0.714; sensitivity, 0.976) in training set and 0.895 with 95% CI, 0.777–1.000 (accuracy, 0.778; specificity, 0.887; sensitivity, 0.772) in testing set. The ADC-based model had an AUC of 0.790 with 95% CI: 0.794–0.945 (accuracy, 0.774; specificity, 0.714; sensitivity, 0.976) in training set and 0.796 with 95% CI, 0.777–1.000 (accuracy, 0.778; specificity, 0.889; sensitivity, 0.772) in testing set. The combined model integrating T2 and ADC features showed an AUC of 0.908 with 95% CI: 0.845–0.971 (accuracy, 0.857; specificity, 0.762; sensitivity, 0.952) in training set and 0.926 with 95% CI: 0.813-1.000 (accuracy, 0.852; specificity, 1.000; sensitivity, 0.778) in testing set. Calibration curve showed that the combined score had a good calibration degree, and the decision curve demonstrated that the combined score was of benefit for clinical use.

**Data Conclusion:**

Radiomics analysis of T2W and ADC images showed significant relevance in the prediction of microsatellite status, and the accuracy of combined model of ADC and T2W features was better than either alone.

## Introduction

The incidence rate of rectal cancer patients was the third and the mortality rate was the fourth in malignant tumors (1). The 5-year disease-free survival rate of colorectal cancer was associated with MIS status (2). Colorectal cancer patients with the MSI has a better prognosis than with MSS, meaning that MSI status can be used as a good prognostic indicator (3, 4). Patients with MSI only benefit from the 5-FU–based adjuvant chemotherapy, but the rectal cancer with MSS does not respond to 5-FU–based neoadjuvant therapy (5–7). Some patients with dDDM/MSI tumor respond to immune checkpoint inhibitors (ICI) therapy (8). Therefore, the microsatellite status of colorectal cancer is helpful to the selection of neoadjuvant therapy and predicts the prognosis of colorectal cancer. The status of MSI is assessed using colonoscopy biopsy. However, this method had two challenges. First, the DNA extracted from the sample obtaining from colonoscopy biopsy may not meet the minimum quality criteria from the genetic assay; second, because of the high heterogeneity of colorectal cancer, the accurate MSI status of colorectal cancer cannot be obtained by puncture pathology examination (9). Therefore, we need to find a way to preoperatively predict MSI status and reflect heterogeneity of rectal cancer.

Now, there is a certain correlation between the texture parameters extracted from tumor images and the gene expression of tumors. CT image texture parameters of colorectal cancer have certain correlation with KRAS/NRAS/BRAF gene mutations in colorectal cancer, and the texture parameters extracted from MRI images can help predict TCGA/TCIA molecular subtypes in breast cancer (10, 11). We hypothesize that the MR imaging radiomics parameter modeling can predict the MSI statue of rectal cancer. Therefore, the purpose of this study is to assess the performance of predicting the rectal cancer MSI status used MR radiomics.

## Materials and Methods

### Patients

**Figure 1:**
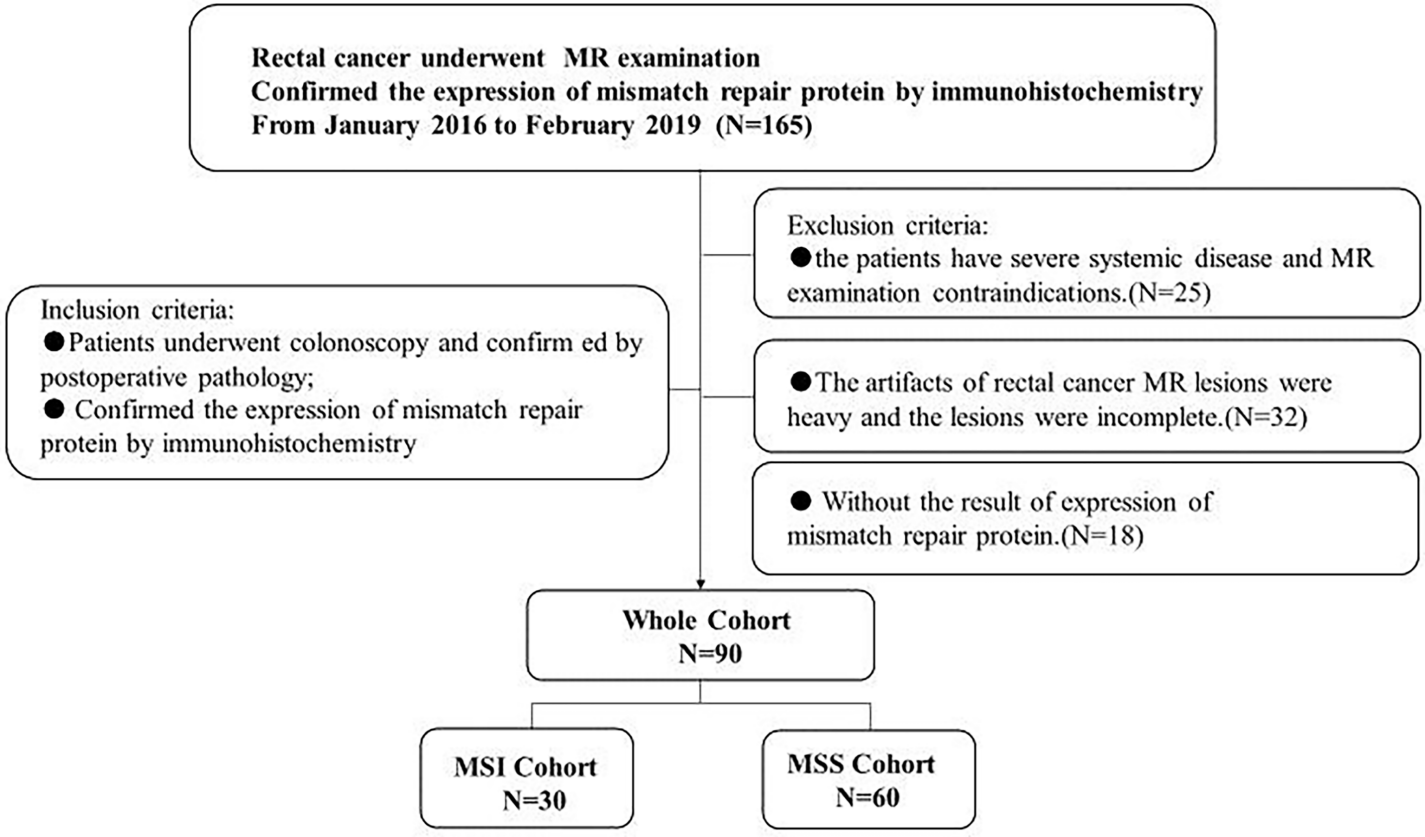
Flow diagram of patient selection.

This prospective study was approved by the institutional ethical committee of our hospital, and all patients signed a written informed consent prior to MR. From January 2016 to February 2019, 90 patients who met the following criteria were included in our study. Inclusion criteria: (1) patients who were highly suspected of rectal cancer underwent colonoscopy and confirmed by postoperative pathology; (2) IHC examination was performed to determine the expression status of rectal cancer mismatch repair protein. Exclusion criteria: (1) the patients have severe systemic disease and MR examination contraindications. (2) The artifacts of rectal cancer MR lesions were heavy, and the lesions were incomplete. (3) Without the result of expression of mismatch repair protein. The details of the patient selection are shown in **Figure 1**.

### Determination of MSI Statues

Specimens were fixed with 4% formaldehyde, embedded in paraffin, serially sectioned at 4 μm, and stained with conventional HE. Immunohistochemical staining was performed using the EnVision two-step method. The primary antibody was rabbit anti-human MLH1, MSH2, MSH6, PMS2. DAB coloration, hematoxylin counterstaining. Nuclear staining of tumor tissue stromal cells and normal non-tumor mucosa was used as a positive control; antibody diluent was used instead of an antibody as a negative control. In the area where the nuclear staining is good in the internal control (normal intestinal glandular epithelium and interstitial cells, etc.), the tumor cell nuclear staining is positive, and the coloration is negative, and if the protein expression is negative, it is assumed that there is loss of mismatch repair protein expression, meaning that the tumor is MSI.

### MR Image Acquisition, Preprocessing, and Segmentation

Standardized MR scans were performed with a 3.0T scanner (Siemens skyra3.0T, Germany): all the patients underwent routine MR and multiple b-value DW sequence scans. The position of the tumor was determined by sagittal T2W-TSE sequence image, and the T2W image perpendicular to the rectal axis (TR/TE, 3380/95 ms; field of view [FOV], 210 mm × 210 mm; voxel size, 0.7 mm × 0.7 mm × 3.0 mm), DW sequence image (voxel size, 2.4 mm × 2.4 mm × 3.0 mm; b values, 50, 800) and ADC image were automatically generated.

All the T2W and ADC images were uploaded to A.K software (Artificial Intelligence Kit; A.K., GE Healthcare, China) for histogram matching and homogenization.

**Figure 2:**
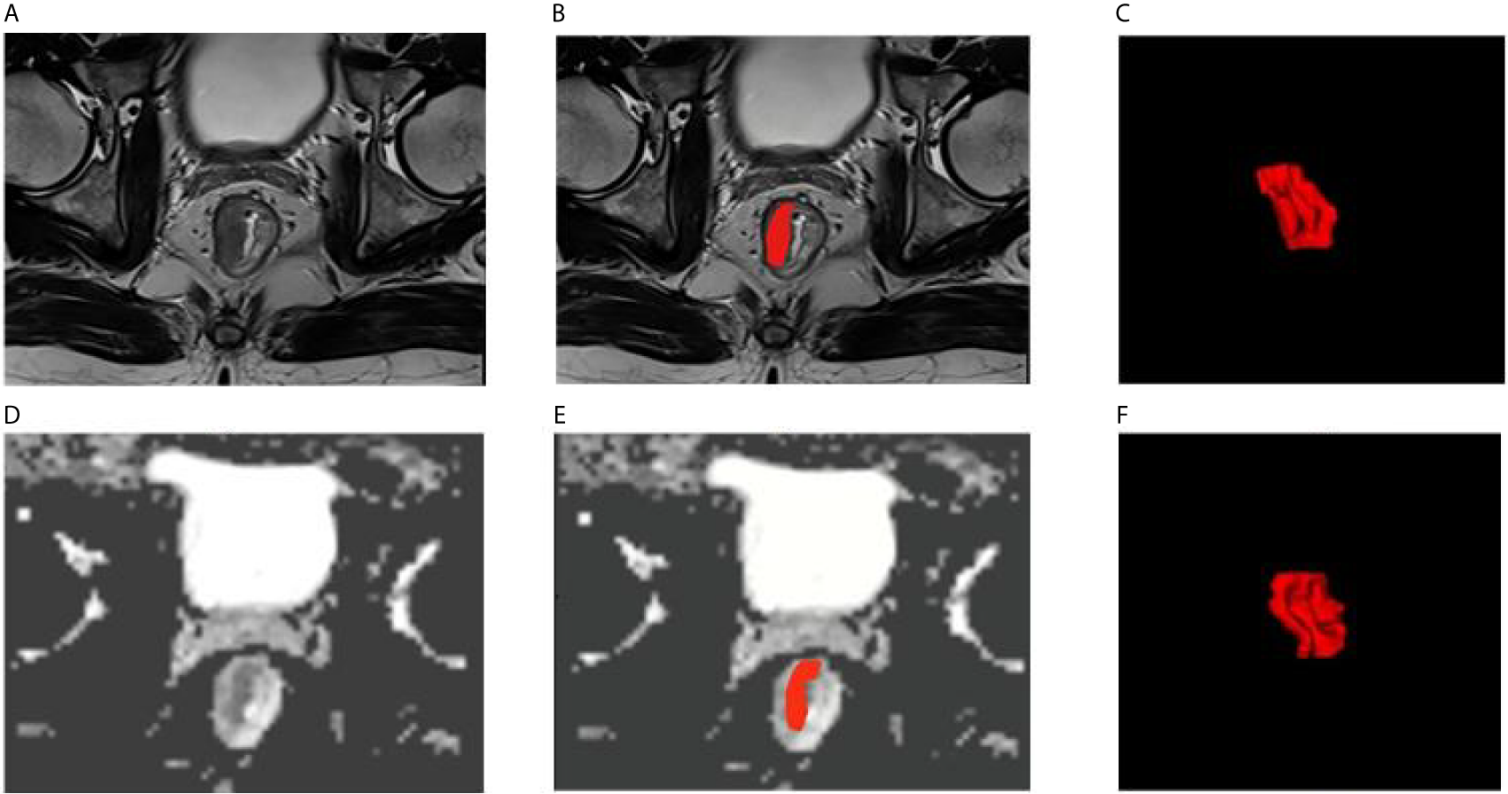
Examples of rectal cancer segmentation. **(A–C)** T2WI images, **(D–F)** ADC images, **(C, F)** 3D view.

Two radiologists with experience of 15 and 30 years used the open-source ITK-SNAP software (version 3.6.0 Apr 1, 2017, Copyright(c) 1998-2017 Paul A. Yushkevich Guido Gerig) to manually delineated the ROI of the tumor in the T2W image and the ADC image with the patient’s colonoscopy results, they sketch the lesions layer by layer from the edge of the tumor independently, and then generated the 3D-ROI (**Figure 2**).

### Radiomics Features Extraction

**Figure 3:**
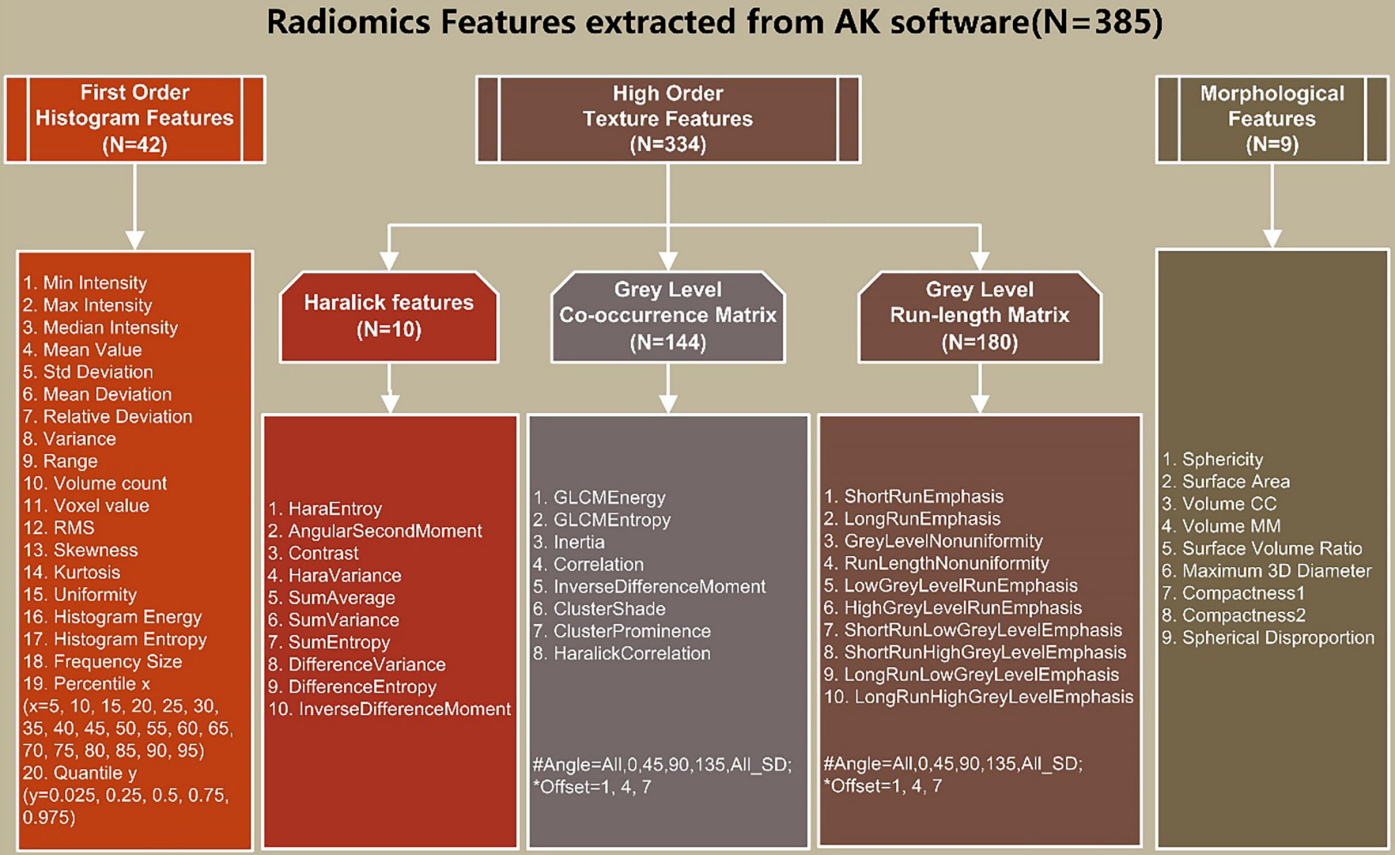
Details of extracted radiomics features: (1) First-order histogram features (*n* = 42); (2) high-order texture features (n=334): Haralick features (n=10), gray level co-occurrence matrix (GLCM) (n=144), and gray-level run-length matrix (GLRLM) (*n* = 180); (3) morphological features (*n* = 9).

All images and ROIs were uploaded to AK software and then 385 features were automatically calculated, as shown in **Figure 3**.

### Features Selection and Modeling

First, in order to assess the inter-observer reproducibility of the radiomics features so as to establish a robust model, calculated the intra-class coefficient correlation (ICC) index. The features of ICC ≥ 0.75 were retained, which yielded a relatively high intra-observer stability in the segmented tumor volume, and the features of ICC< 0.75 were abandoned.

Then, the remaining stable features were subjected to random forest model and the top 5 of important index were retained, and the rest were discarded. Finally, multivariate logic stepwise regression was used to remove meaningless features backward and construct a radiomics score, which called ADC score and T2 score.

To generate an integrated model, ADC score and T2 score were combined using multivariate logistic regression analysis, after which, a combined score was constructed.

### Balancing Data Samples

We randomly divided the proportion of these 90 patients into 7:3 into training and testing sets. Considering that logistic regression performs best when case-to-noncase ratio is 1:1, the number of patients with MSI was smaller than the number of patients with MSS, and the sample imbalance would have an adverse impact on the performance of a classifier, Thus, we used the synthetic minority oversampling technique (SMOTE) (12) in the training set to increase the weight of minority samples and to balance the samples of patients, where the ratio of MSI and MSS was 1:1. However, we did not apply the SMOTE in testing set mainly because: firstly the bias data set was independent from the training set, so it had no effect at modeling, and second, the incidence of MSI was lower than MSS in the real world, so we use the real testing set to obtain a real testing result of the model. In the end, we randomly selected one tenth of the patients with MSI and patients with MSS to test the model.

### Statistical Analysis

**Figure 4:**
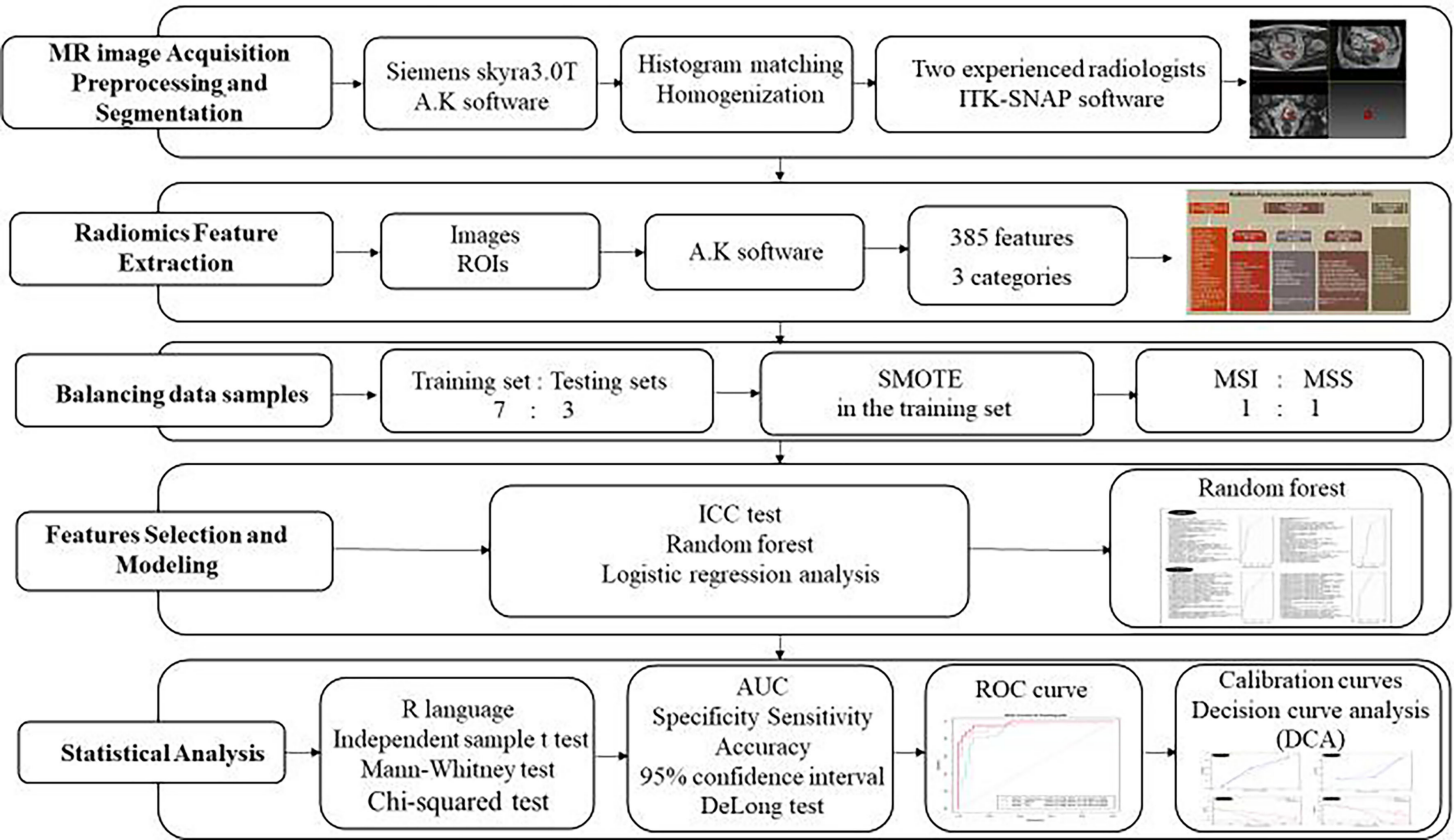
Flow diagram of our study.

All the statistical analyses were performed with R language (Version 1.0.143- ^©^2009-2016 R Studio, Inc.). The differences in patient features between patients with MSI and MSS were assessed by the independent-sample *t* test or Mann-Whitney test according to the data distribution type. The chi-squared test was used to compare the significance of the differences between categorical variables. Receiver operating characteristic (ROC) curves were plotted to evaluate the diagnostic performance of the radiomics score in both training and testing sets. Area under the ROC curve (AUC) with 95% confidence interval (CI), specificity, sensitivity, and accuracy were calculated. DeLong test was used to compare the differences of AUC values between different models in the training and testing set. To evaluate whether the models were well-calibrated or not, calibration curves were plotted in both training and testing sets. Decision curve analysis (DCA) was conducted to determine the clinical usefulness of the models by quantifying the net benefits at different threshold probabilities in both training and testing sets. The flowchart of our study was shown in **Figure 4**.

## Results

### Clinical Characteristics

In our research, 60 patients were MSS (38 males, 22 females; mean age, 61.12; age range, 31–82 years) and 30 patients were MSI (16 males, 14 females, average age, 60.29 years; age range, 30–83 years. Our results showed that there was no significant difference in clinical features between MSI and MSS patients with age, gender, tumor location, size, differentiation degree, TNM stage, hypertension, diabetes, family history of cancer, and smoking and alcohol history. The details of baseline characteristics results were shown in **Table 1**.

**Table 1:** The results of baseline characteristics.

Characteristic		MSS (N=60)	MSI (N=30)	P values
**Age, mean ± SD (years)**		61.03 ± 11.23	58.05 ± 10.55	0.252
**Gender, n (%)**	**Male**	38(63.3)	15(50.0)	0.226
	**Female**	22(36.7)	15(50.0)	
**Tumor location, n (%)**	**High**	8(13.3)	4(13.3)	0.983
	**Medium**	39(65.0)	19(63.3)	
	**Low**	13(21.7)	7(23.4)	
**Length of tumor, mean ± SD (cm)**		3.97 ± 1.81	4.46 ± 1.16	0.334
**Differentiation, n (%)**	**Well**	3(5.0)	0(0.0)	0.159
	**Moderately**	43(71.7)	17(56.7)	
	**Poorly**	10(16.7)	8(26.7)	
	**Mucinous**	4(6.6)	5(16.6)	
**Stages, n (%)**	**I**	22(36.7)	8(26.7)	0.606
	**II**	14(23.3)	9(30.0)	
	**III**	24(40.0)	13(43.3)	
	**IV**	0(0.0)	0(0.0)	
**T-staging, n (%)**	**T1**	4(6.7)	0(0.0)	0.434
	**T2**	23(38.3)	10(33.3)	
	**T3**	32(53.3)	19(63.3)	
	**T4**	1(1.7)	1(3.4)	
**N-staging, n (%)**	**N0**	35(58.3)	17(56.7)	0.88
	**N+**	25(41.7)	13(43.3)	
**Hypertension, n (%)**	**Yes**	17(28.3)	8(26.7)	0.868
	**No**	43(71.7)	22(73.3)	
**Family history, n (%)**	**Yes**	0(0.0)	0(0.0)	1.000
	**No**	60(100.0)	30(100.0)	
**Diabetes mellitus, n (%)**	**Yes**	7(11.7)	7(23.3)	0.150
	**No**	53(88.3)	23(76.7)	
**Smoke, n (%)**	**Yes**	18(30.0)	9(30.0)	1.000
	**No**	42(70.0)	21(70.0)	
**Drink, n (%)**	**Yes**	11(18.3)	6(20.0)	0.849
	**No**	49(81.7)	24(80.0)	

### Feature Selection and Radiomics Models Building

After consistency analysis, a total of 203 and 89 radiomics features, respectively, extracted from T2W and ADC images had ICC values of more than 0.75 and were then used for features selection and modeling.

**Figure 5:**
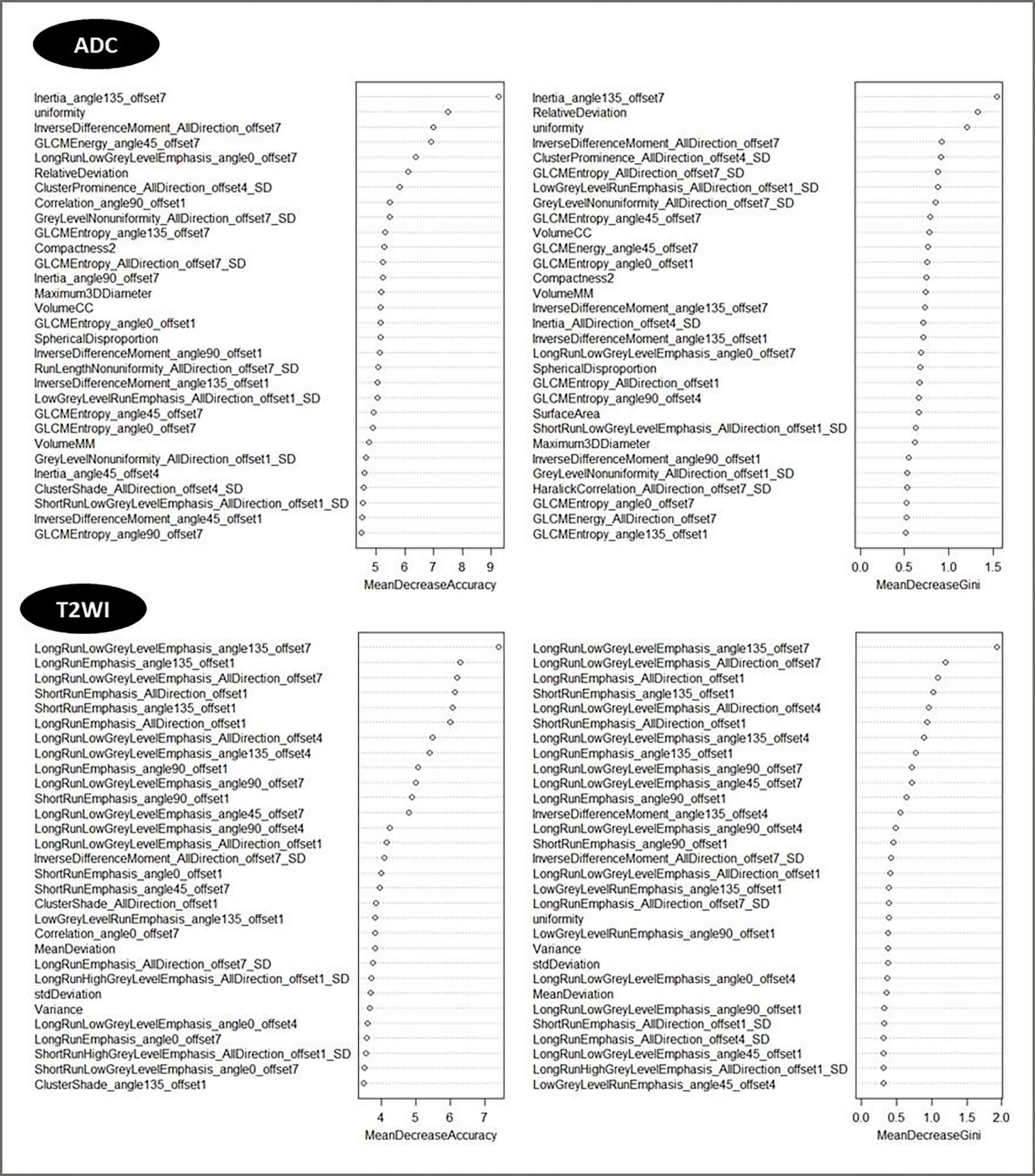
The importance rank of features in T2W and ADC images.

Then top 5 features based on T2W and ADC imaging, which had the biggest mean decrease accuracy value in the random forest model, were obtained. According to their importance, the features were ranked as shown in **Figure 5**.

The five selected features were then entered into multivariate logistic regression using backward elimination method, after which, a total of two and three features were retained respectively from the T2W and ADC images for the final modeling. Radiomics scores (Rad-scores) were calculated for each patient *via* a linear combination of the selected features that were weighted by their respective coefficients. Combined score was calculated *via* a linear combination of the ADC score and T2 score. The calculation formula was as follows:


$$\begin{array}{l} \text{T}2\;\text{Score}=773+991*\\ \text{LongRunLowGreyLevelEmphasis}\_\text{AllDirection}\_\text{offset}7-777*\\ \text{ShortRunEmphasis}\_\text{angle}135\_\text{offset}1. \end{array}$$

$$\begin{array}{l} \text{ADC}\;\mathrm{Score}=-5.48+1.4*10^{-3}\;*\;\text{RelativeDeviation}+8.33*\;\text{uniformity}+0.72\\ {}*\text{LongRunLowGreyLevelEmphasis}\_\text{angle}0\_\text{offset}7. \end{array}$$

Combine Score=0.092+1.17*T2 Score+0.92*ADC Score.

### Performance of the Radiomics Models

**Figure 6:**
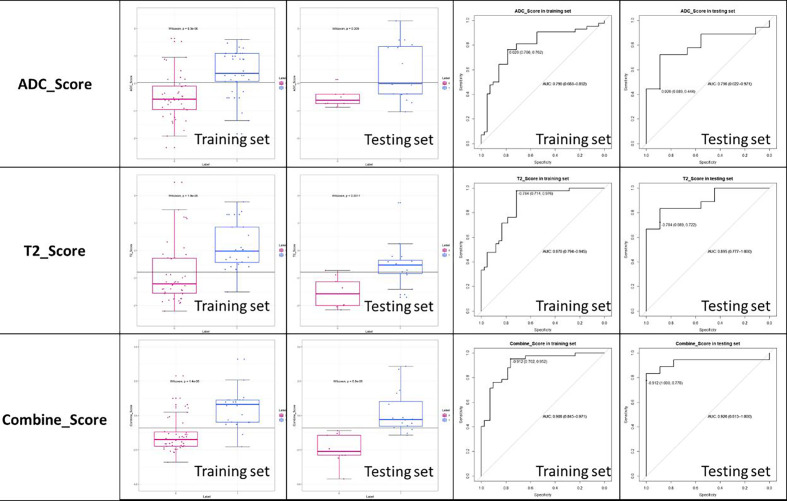
The box-plot and ROC curve of ADC score, T2 score, and combined score.

**Figure 7:**
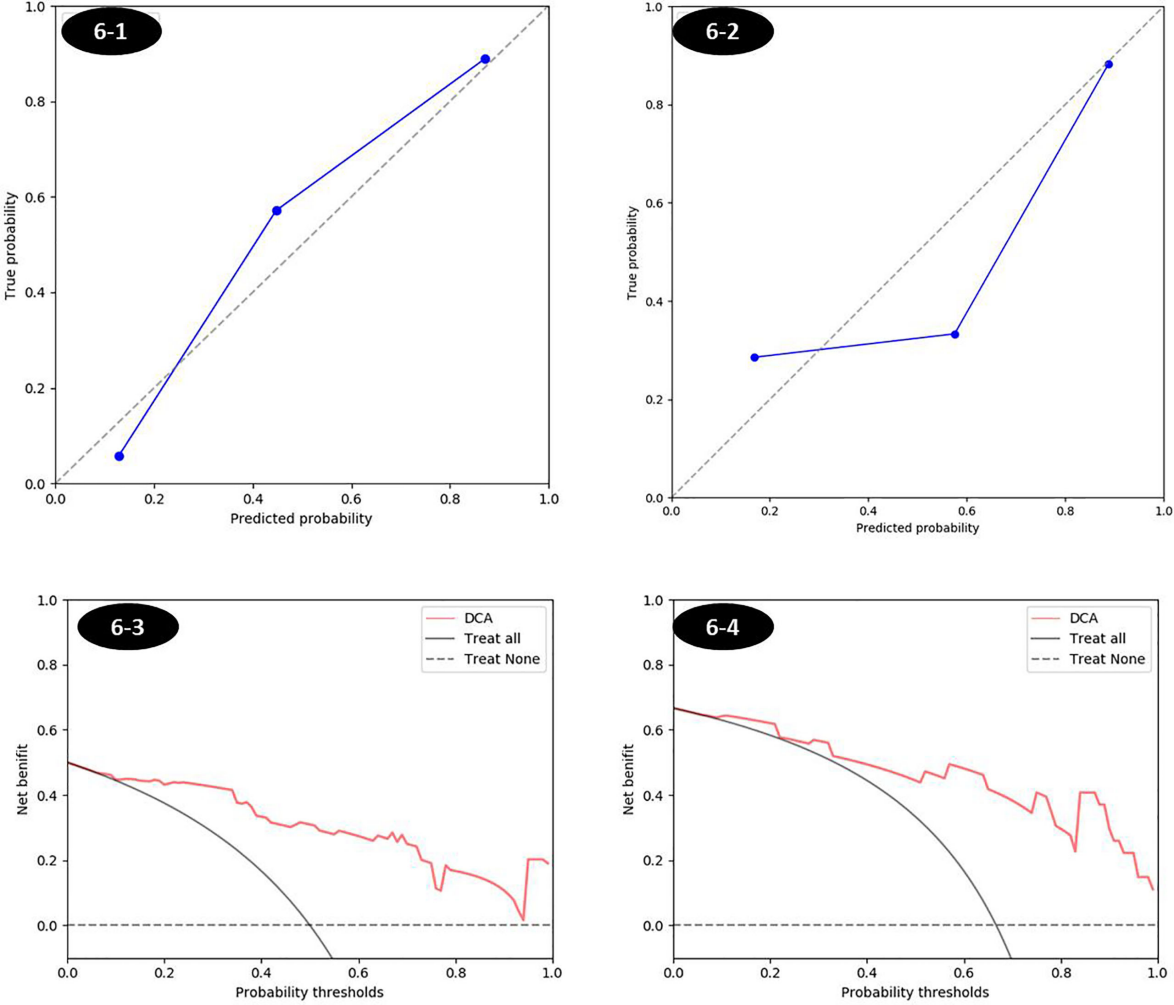
The calibration curves and decision curves of combined score in training and testing sets. (1) Calibration curve was shown in Figures 6-1 and 6-2, respectively, for training and testing sets. The closer to the diagonal reference line, the better the fitness of the model. (2) The decision curve in Figures 6-3 and 6-4 was for the combined score in training and testing set respectively, which showed that whatever the threshold probability was, using the combined score to predict the MSI status added more benefit than either the treat-all scheme (assuming all lesions are MSI) or the treat-none scheme (assuming all lesions are MSS).

The established T2 score, ADC score, and combined score were significantly associated with the status of MSI in both training and testing sets. The scores were all significantly higher in MSI group than that in MSS group for both training and testing sets. The box plot was shown in **Figure 6**. The performance was better for combined score with an AUC of 0.908 (95% CI, 0.845–0.971) in training set and 0.926 (95% CI, 0.813–1.0) in testing set than either one, where T2 score showed an AUC of 0.870 (95% CI: 0.794-0.945) in training set and 0.895 (95% CI: 0.777-1.000) in testing set and the ADC score showed an AUC of 0.790(95% CI: 0.794-0.945) in training set and 0.796 (95% CI: 0.622-0.971) in testing set. The accuracy, sensitivity, and specificity based on the optimal cutoff value for all the models were shown in **Table 2** and **Figure 6**. Delong test demonstrated that there was no significant difference between the performance of the model in training and testing set, which showed the relative robustness of the model. The calibration curve showed that the combined model fitted well, and the decision curve analysis showed the clinical usefulness of the combined model, as shown in **Figure 7**.

**Table 2:** Performance of the Rad-Scores established based on T2W, ADC Images and both combination for MSI and MSS Discrimination.

Sequence	Data Set	AUC	95% CI	ACC	Specificity	Sensitivity	Cutoff	P values
								(DeLong test)
**T2WI**	Train	0.870	0.794–0.945	0.845	0.714	0.976	0.314	0.724
	Test	0.895	0.777–1.000	0.778	0.887	0.772	0.314	
**ADC**	Train	0.790	0.794–0.945	0.774	0.714	0.976	0.507	0.949
	Test	0.858	0.738–0.978	0.722	0.889	0.556	0.507	
**T2WI+ADC**	Train	0.908	0.845–0.971	0.857	0.762	0.952	0.290	0.782
	Test	0.926	0.813–1.000	0.852	1.000	0.778	0.290	

## Discussion

Our study revealed that the models of radiomics features based on T2W and ADC imaging can preoperatively predict the MSI status efficiently. The prediction performance of combined score (T2 and ADC) has a higher degree of specificity than T2 score or ADC score.

CT can also be used as a technique to determine MSI status, all the measurements (NICA, V, D; kP, A, V, D; Eff-Z) of MSI based on energy spectrum CT were significantly lower, where MSS and AUC of multivariate logistic regression analysis was 0.886 (13). In our research, the combined score with an AUC of 0.908 (95% CI: 0.845–0.971) in the training set and 0.926 (95% CI: 0.813–1.0) in the testing set, which was better than CT. The reason might be that different sequences of MR represent the signal strength and quantitative parameters and could reflect heterogeneity of rectal cancer. Moreover, because of the presence of radiation in CT examination, patients with colorectal cancer who received neoadjuvant therapy and underwent multiple reexaminations may receive more radiation dose, which could become a factor in influencing the outcome.

Different tumor histology showed different characteristics of radiomics based on tumor images, the difference in texture and histogram between MSI and MSS of rectal cancer indicated the different grayscale distribution of the two images. The histogram feature described the overall distribution of grayscale in the ROI of image (14) and the texture feature described the distribution of local grayscale values of the image (15). ADC images could reflect the degree of diffusion of extracellular water molecules and the ADC value of malignant tumors was significantly higher than that of benign tumors, which was due to the rapid growth of malignant tumor cells, small extracellular space, and limited movement of water molecules (16). Because of the loss of mismatch repair protein gene (17), tumor cells showed relative poor differentiation and relatively more mucinous tissue and necrotic components exist (18), more CD3+, CD4+, CD8+, CD20+, and CD68+ cell infiltration and showed higher microvessel density (MVD) (19), resulting in different image grayscale distribution, which caused differences in image histogram features and texture features. Therefore, they could reflect certain spatial characteristics and could predict the MSI status. Meanwhile, T2W images formed by the signal intensity were susceptible to the MR field strength, excitation times, and noise. ADC image consists of ADC values at different locations in the image. The different histological states in the tissues and cells of colorectal cancer may lead to different extracellular space and imaging signal intensity, resulting in the combination of T2 score and ADC score, which had a better prediction effect than T2W Score or ADC image score alone.

To avoid the influence of the difference between the scanning parameters of t2w and ADC images, we performed image histogram matching and homogenization processing first to make the contrast of each image consistent, ensuring that the extracted features were credible before delineating the ROI. Second, we performed a consistency test on the features extracted by two radiologists and retained the features with a consistency coefficient of >0.75 to reduce the error caused by the physician manually delineating. In our study, because of the presence of 30 MSI patients and 60 MSS patients, there was an inter-group data imbalance. Therefore, we used the SMOTE method to strike a balance with the MSI oversampling. At the same time, we also calculated the results without amplification in the MSI group, suggesting that the T2W and ADC model significantly improved after using the SMOTE method. This shows that the performance of the radiomics model can be further improved by using the balanced data set.

Our research currently had some limitations. First, only 90 patients were enrolled, and the number of patients needed to be increased. Second, our study used immunohistochemistry to detect the expression of mismatch repair proteins to determine the MSI status of rectal cancer; however, a study has shown that some colorectal cancers may lose MSH6 gene inactivation after receiving neoadjuvant therapy, and resulting in the loss of MSH6 protein expression, which would cause a certain degree of inaccurate in immunohistochemistry results (20). This part of the patients had better undergo PCR to determine the true microsatellite status. In our study, there were two patients with MSH6 deletion, but no PCR test was conducted, which may affect the experimental results.

## Data Availability Statement

The raw data supporting the conclusions of this article will be made available by the authors, without undue reservation.

## Ethics Statement

The studies involving human participants were reviewed and approved by The Institutional Ethical Committee of Jilin University. Written informed consent for participation was not required for this study in accordance with the national legislation and the institutional requirements. The animal study was reviewed and approved by The Institutional Ethical Committee of Jinlin University.

## Author Contributions

ZL: methodology, resources, and data curation. HD: methodology, conceptualization, and investigation. YL: conceptualization and supervision. FP: writing - original draft and data curation. YY: resources and data curation. MZ: project administration, supervision, and funding acquisition. All authors contributed to the article and approved the submitted version.

## Funding

This study was financially supported by the key project grant from the Natural Science Foundation of Jilin Province, China (No. 20180101125JC), the Key Project of Social Development Fund of Science and Technology Department of Jilin Province, China (No. 20200403129SF), the National Natural Science Foundation of China, 81971573 to HD, the Suzhou Gusu Medical Youth Talent, GSWS2020019 to HD, and the 13th Five-Year Science and Technology Project of the Education Department of Jilin Province, JJKH20190064KJ to MZ.

## Correction note

A correction has been made to this article. Details can be found at: 10.3389/fonc.2026.1841797.

## Conflict of Interest

The authors declare that the research was conducted in the absence of any commercial or financial relationships that could be construed as a potential conflict of interest.

## Abbreviations

MR, magnetic resonance; MSI, microsatellite instability; MSS, microsatellite stable; ROI, region of interest; AUC, area under the curve; ICC, intraclass correlation coefficient; SMOTE, synthetic minority oversampling technique; ROC, receiver operating characteristic; DCE-MRI, dynamic contrast-enhanced magnetic resonance imaging; MVD, micro-vessel density.

## References

[1] Li D Li Q He Y. Epidemiology of Colorectal Cancer. Cancer Prev Treat (2015) 42(03):305–10. doi: 10.3971/j.issn.1000-8578

[2] Ribic CM Sargent DJ Moore MJ Thibodeau SN French AJ Goldberg RM. Tumor Microsatellite-Instability Status as a Predictor of Benefit From Fluorouracil-Based Adjuvant Chemotherapy for Colon Cancer. N Engl J Med (2003) 349(3):247–57. doi: 10.1056/NEJMoa022289 PMC3584639 12867608

[3] Popat S Hubner R Houlston RS. Systematic Review of Microsatellite Instability and Colorectal Cancer Prognosis. J Clin Oncol (2005) 23(3):609–18. doi: 10.1200/JCO.2005.01.086 15659508

[4] Robert G Hyeja K Hsieh ET Aronson MD Holowaty EJ Bull SB. Tumor Microsatellite Instability and Clinical Outcome in Young Patients With Colorectal Cancer. N Engl J Med (2000) 342(2):69–77. doi: 10.1056/NEJM200001133420201 10631274

[5] Sargent DJ Silvia M Genevieve M Thibodeau SN Roberto L Hamilton SR. Defective Mismatch Repair as a Predictive Marker for Lack of Efficacy of Fluorouracil-Based Adjuvant Therapy in Colon Cancer. J Clin Oncol (2010) 28(20):3219–26. doi: 10.1200/JCO.2009.27.1825 PMC2903323 20498393

[6] Du C Zhao J Xue W Dou F Gu J. Prognostic Value of Microsatellite Instability in Sporadic Locally Advanced Rectal Cancer Following Neoadjuvant Radiotherapy. Histopathology (2013) 62(5):723–30. doi: 10.1111/his.12069 23425253

[7] Demes M Scheil-Bertram S Bartsch H Fisseler-Eckhoff A. Signature of Microsatellite Instability, KRAS and BRAF Gene Mutations in German Patients With Locally Advanced Rectal Adenocarcinoma Before and After Neoadjuvant 5-FU Radiochemotherapy. J Gastrointestinal Oncol (2013) 4(2):182–92. doi: 10.3978/j.issn.2078-6891.2013.012 PMC3635176 23730514

[8] Mike N Sam TS Marissa L Elizabeth L Amy D Hans P. An Update on the Use of Immunotherapy in Patients With Colorectal Cancer. Expert Rev Gastroenterol Hepatol (2021) 15(3):291–304. doi: 10.1080/17474124.2021.1845141 33138649

[9] Gado A Ebeid B Abdelmohsen A Axon A. Improving the Yield of Histological Sampling in Patients With Suspected Colorectal Cancer During Colonoscopy by Introducing a Colonoscopy Quality Assurance Program. Gastroenterol Res (2011) 4(4):157–61. doi: 10.4021/gr334w PMC5139727 27942333

[10] Yang L Dong D Fang M Zhu Y Zang Y Liu Z. Can CT-Based Radiomics Signature Predict KRAS/NRAS/BRAF Mutations in Colorectal Cancer? Eur Radiol (2018) 28(5):2058–67. doi: 10.1007/s00330-017-5146-8 29335867

[11] Li H Zhu Y Burnside E Huang E Drukker K Hoadley K. Quantitative MRI Radiomics in the Prediction of Molecular Classifications of Breast Cancer Subtypes in the TCGA/TCIA Data Set. NPJ Breast Cancer (2016) 2(1):9–29. doi: 10.1038/npjbcancer.2016.12 PMC5108580 27853751

[12] Fetit A Novak J Rodriguez D Auer D Clark C Grundy R. Radiomics in Paediatric Neuro-Oncology: A Multicentre Study on MRI Texture Analysis. NMR Biomed (2018) 31(1):3781. doi: 10.1002/nbm.3781 29073725

[13] Wu J Lv Y Wang N Zhao Y Zhang P Liu Y. The Value of Single-Source Dual-Energy CT Imaging for Discriminating Microsatellite Instability From Microsatellite Stability Human Colorectal Cancer. Eur Radiol (2019) 29(7):3782–90. doi: 10.1007/s00330-019-06144-5 30903331

[14] Just N. Improving Tumour Heterogeneity MRI Assessment With Histograms. Br J Cancer (2014) 111(12):2205–13. doi: 10.1038/bjc.2014.512 PMC4264439 25268373

[15] Guan Y Li W Jiang Z Zhang B Chen Y Huang X. Value of Whole-Lesion Apparent Diffusion Coefficient (ADC) First-Order Statistics and Texture Features in Clinical Staging of Cervical Cancers. Clin Radiol (2017) 72(11):951–8. doi: 10.1016/j.crad.2017.06.115 28728757

[16] Song Y Yoon Y Chong Y Seo S Choi Y Sohn I. Diagnostic Performance of Conventional MRI Parameters and Apparent Diffusion Coefficient Values in Differentiating Between Benign and Malignant Soft-Tissue Tumours. Clin Radiol (2017) 72(8):691.e1–.e10. doi: 10.1016/j.crad.2017.02.003 28274509

[17] Boland C Goel A. Microsatellite Instability in Colorectal Cancer. Gastroenterology (2010) 138(6):2073–87.e3. doi: 10.1053/j.gastro.2009.12.064 20420947 PMC3037515

[18] Gupta R Sinha S Paul R. The Impact of Microsatellite Stability Status in Colorectal Cancer. Curr Prob Cancer (2018) 42(6):548–59. doi: 10.1016/j.currproblcancer.2018.06.010 30119911

[19] De Smedt L Lemahieu J Palmans S Govaere O Tousseyn T Van Cutsem E. Microsatellite Instable vs Stable Colon Carcinomas: Analysis of Tumour Heterogeneity, Inflammation and Angiogenesis. Br J Cancer (2015) 113(3):500–9. doi: 10.1038/bjc.2015.213 PMC4522625 26068398

[20] Kuan S Ren B Brand R Dudley B Pai R. Neoadjuvant Therapy in Microsatellite-Stable Colorectal Carcinoma Induces Concomitant Loss of MSH6 and Ki-67 Expression. Hum Pathol (2017) 63:33–9. doi: 10.1016/j.humpath.2017.02.003 28232158

