# Corrigendum: Radiomics Analysis of Multi-Sequence MR Images For Predicting Microsatellite Instability Status Preoperatively in Rectal Cancer

**DOI:** 10.3389/fonc.2021.781636

**Published:** 2021-10-11

**Authors:** Zongbao Li, Hui Dai, Yunxia Liu, Feng Pan, Yanyan Yang, Mengchao Zhang

**Affiliations:** ^1^ China-Japan Union Hospital of Jilin University, Changchun, China; ^2^ The First Affiliated Hospital of Soochow University, Suzhou, China

**Keywords:** magnetic resonance, rectal cancer, microsatellite instability, radiomics, multi-sequence MR

In the original article, we neglected to include the funder the National Natural Science Foundation of China, 81971573 to HD, the Suzhou Gusu Medical Youth Talent, GSWS2020019 to HD, and the 13th Five-Year Science and Technology Project of the Education Department of Jilin Province, JJKH20190064KJ to MZ.

In the published article, there was an error regarding the Affiliation for Hui Dai. Instead of “China-Japan Union Hospital of Jilin University, Changchun, China”, it should be “The First Affiliated Hospital of Soochow University, Suzhou, Jiangsu, China”.

The authors apologize for these errors and state that this does not change the scientific conclusions of the article in any way. The original article has been updated.

## Publisher’s Note

All claims expressed in this article are solely those of the authors and do not necessarily represent those of their affiliated organizations, or those of the publisher, the editors and the reviewers. Any product that may be evaluated in this article, or claim that may be made by its manufacturer, is not guaranteed or endorsed by the publisher.

