# Correction: Radiomics analysis of multi-sequence MR images for predicting microsatellite instability status preoperatively in rectal cancer

**DOI:** 10.3389/fonc.2026.1841797

**Published:** 2026-05-14

**Authors:** Zongbao Li, Hui Dai, Yunxia Liu, Feng Pan, Yanyan Yang, Mengchao Zhang

**Affiliations:** 1 China-Japan Union Hospital of Jilin University, Changchun, China; 2 The First Affiliated Hospital of Soochow University, Suzhou, China

**Keywords:** magnetic resonance, rectal cancer, microsatellite instability, radiomics, multi-sequence MR

“First, in order to assess the intra-observer reproducibility of the radiomics features so as to establish a robust model, calculated the intra-class coefficient correlation (ICC) index.” was incorrect.

A correction has been made to the section **Materials and methods**, *Features selection and modeling*:

“First, in order to assess the inter-observer reproducibility of the radiomics features so as to establish a robust model, calculated the intra-class coefficient correlation (ICC) index.”

The original version of this article has been updated.

